# E-Cigarettes and Associated Health Risks: An Update on Cancer Potential

**DOI:** 10.3390/arm91060038

**Published:** 2023-11-14

**Authors:** Rakesh Sahu, Kamal Shah, Rishabha Malviya, Deepika Paliwal, Sakshi Sagar, Sudarshan Singh, Bhupendra G. Prajapati, Sankha Bhattacharya

**Affiliations:** 1 Department of Pharmacy, School of Medical and Allied Sciences, Galgotias University, Greater Noida 201310, India; rishabha.malviya@galgotiasuniversity.edu.in (R.M.); deepika14paliwal@gmail.com (D.P.); sakshisagar312@gmail.com (S.S.); 2 Department of Pharmaceutical Chemistry, Institute of Pharmaceutical Research, GLA University, Mathura 281406, India; kamal0603@gmail.com; 3 Department of Pharmaceutical Sciences, Faculty of Pharmacy, Chiang Mai University, Chiang Mai 50200, Thailand; sudarshan.s@cmu.ac.th; 4 Shree S. K. Patel College of Pharmaceutical Education and Research, Ganpat University, Kherva 384012, India; 5 Department of Pharmaceutics, School of Pharmacy & Technology Management, SVKM’S NMIMS Deemed-to-Be University, Shirpur 425405, India; sankhabhatt@gmail.com

**Keywords:** cancer, e-cigarette, generation of e-cigarette, chemicals in e-cigarette, flavors of e-cigarette

## Abstract

**Highlights:**

**What are the main findings?**

- Due to the possibility of dangerous chemicals and flavorings in the aerosol, there is evidence to suggest that using e-cigarettes may raise the risk of cancer as well as other ailments including cardiovascular and respiratory disease It’s crucial to use caution when utilizing e-cigarettes and to think about other options for quitting smoking.

**What is the implication of the main finding?**

- Although there may not be many advantages to using e-cigarettes to help people quit smoking, users should carefully examine the potential risks involved before using them, including the possibility of developing cancer.
- The long-term cancer risk linked to e-cigarette usage is yet unknown. On the other hand, the review will provide the investigator with more recent information on e-cigarettes.

**Abstract:**

The potential cancer risk associated with electronic-cigarette (e-cigarette) use is ongoing and remains a subject of debate. E-Cigarettes work by heating a liquid that usually contains nicotine, flavorings, and other chemicals. When the liquid is heated, users inhale an aerosol into their lungs. While e-cigarettes are generally considered less harmful than traditional tobacco products, they still contain potentially harmful chemicals, which can damage DNA and lead to cancer. Several studies have investigated the potential cancer risk associated with e-cigarette use, while other studies have suggested that e-cigarette aerosol may contain carcinogenic chemicals that could increase the risk of lung and bladder cancer in humans. However, these studies are limited in their scope and do not provide conclusive evidence. Overall, the long-term cancer risk associated with e-cigarette use remains uncertain, more research is needed to fully understand the potential risks and benefits of e-cigarettes. However, this review will allow the investigator to get more recent updates about e-cigarettes.

## 1. Introduction

Lik Hong, a Chinese chemist from Hong Kong, created the first commercially successful electronic cigarette (e-cigarette) in 2003. This device heats a nicotine solution to release nicotine without the use of tobacco smoke [1]. When heated, a solution comprising nicotine, moisturizers, and flavorings is typically used in e-cigarettes and vaping devices to supply effluent for inhalation [2]. E-cigarettes are popular among young adults because of reports that they can help smokers stop [3]. This could be so because young adults are drawn to and affected by new trends, especially if they believe that new trends would help them kick their bad habit of smoking cigarettes. Additionally, the fact that they come in a variety of tastes makes them the choice of the majority of users [4]. E-cigarettes are also seen as a superior alternative with fewer health concerns by smokers who are unable to quit [5]. The total prevalence of lifetime e-cigarette vaping was 43.7% across many European nations, with 51.3% of men and 40.5% of women [6]. According to several findings, the prevalence of using e-cigarettes or vaping was, respectively, France (25.46%), Mexico (42.22%), China (24.44%), Australia (12.5%), and the United States (13%) [7,8,9,10,11].

According to GLOBOCAN 2020, there were 19.3 million new instances of cancer detected in 2020, and there were around 10 million cancer-related deaths. By 2040, GLOBOCAN projects that there will be 28 million new instances of cancer [12]. Breast cancer is more common in women worldwide (11.7%) than lung cancer (11%), followed by colon cancer (10.1%), prostate cancer (7.3%), and stomach cancer (5.6%). Lung cancer, which accounts for 1.8 million (18%) of all cancer-related fatalities, is the most common type, followed by colorectal cancer (9%), liver (8.3%), stomach (7.7%), and breast cancer in women came in second (6.9%). Men are more likely to develop lung, prostate, and colon cancers than women, who are more likely to develop breast, colon, and lung cancers [13,14]. Additionally, the results of studies showed that using e-cigarettes may also increase the risk of cardiovascular disease and respiratory disease [15,16].

### 1.1. Types of E-Cigarette 

There are several names for e-cigarettes. They go by the names mods, e-hookahs, e-cigs, vapes, vape pens, and tank systems [17]. Each type of e-cigarette has its advantages and disadvantages, and the choice of e-cigarette will depend on the user’s preferences, experience level, and vaping habits. It is important to choose a high-quality e-cigarette from a reputable manufacturer and to follow proper safety guidelines when using the device. There are different types of e-cigarettes [18]:

#### 1.1.1. Electronic Nicotine Delivery Systems (ENDS)

These are e-cigarette devices that have a flavored solution that usually contains nicotine.

#### 1.1.2. Electronic Non-Nicotine Delivery Systems (ENNDS)

These are e-cigarette devices that have a flavored solution that does not contain nicotine.

By heating liquids, these systems produce an aerosol that the user inhales. These “e-liquids” may or may not include nicotine (no tobacco), but they frequently do. They also frequently contain flavors, additives, and substances that are harmful to human health. It is possible to transport compounds such as nicotine, cannabis, flavors, chemicals, and other substances using electronic cigarettes or vaping equipment. These devices are referred to by a variety of names, including electronic cigarettes, vapes, vape pens, dab pens, dab rigs, tanks, mods, and pod mods. known as “juuling” or “vaping”. “Dabbing” pens” are occasionally used to refer to electronic cigarettes or vape goods used for dabbing [18].

### 1.2. Components of E-Cigarettes

An e-cigarette consists of various pieces, including a mouthpiece, sensor, or button that the user presses to turn on the battery’s heating coil, an atomizer or heating coil, and a tank or reservoir. A heating coil is activated when the user inhales through the mouthpiece, turning the e-cigarette solution into an aerosol that is best defined as an aerosol, but is frequently referred to as vapor-liquid (substance) contained in the cartridge. It can be refilled or pre-filled. It is typically used in unison with a nebulizer. Atomizer: An electrical heating element that assists in converting e-liquid into aerosolized microscopic air droplets. E-cigarettes with sensors instead of a power button turn on when the user inhales through them. The sensors need to be turned on in electronic cigarettes, whether they have a power button or not. Battery: The atomizer may be quickly heated to 400 degrees Fahrenheit using this rechargeable lithium-ion battery [19]. Different generations of e-cigarettes are:

#### 1.2.1. First-Generation ECs

First-generation ECs are frequently referred to be tobacco-like devices with fixed low-voltage batteries since they were created to appear and feel like a standard cigarette. Three first-generation atomizers resemble cigarettes: (1) the three-piece original EC type, which has a separate injector, battery, and liquid tank; (2) a one-piece disposable that integrates the nebulizer, liquid reservoir, and battery into a single item [20]; (3) A two-piece device with a merged injector and liquid tank, while the battery is separate.

#### 1.2.2. Second-Generation ECs

Advanced personal vaporizers or mod systems are other names for second-generation electronic cigarettes (ECs) [21]. These gadgets, sometimes known as cigarlike, are intended to be more potent and adaptable than first-generation ECs [22]. Electrical gadgets from the second generation, also known as “clearomisers”, frequently have larger, variable-voltage batteries that are also known as pen-type batteries. A detachable nozzle with a filament encased in a casing that screws into the liquid tank and batteries is a feature of second-generation cleaners. Compared to ECs, which resemble cigarettes, clearomizers are transparent and feature more liquid reservoirs (or containers). Any refill liquid that is readily available at the moment can be used to fill clearomizers [23,24].

#### 1.2.3. Third-Generation ECs

Third-generation ECs are referred to as “Mods” and contain customized batteries that enable the consumer to modify the voltage, capacity, and capacity. Some models also include other capabilities like the capacity to charge a mobile phone [25]. Third-generation ECs’ ability to create enormous clouds of vapor is one of their key benefits, which may be extremely rewarding for seasoned vapers. Users must be careful not to overdo it because this also means that they can give a very high dose of nicotine [26].

#### 1.2.4. Fourth Generation ECs

Because this generation is evolving quickly and contains many new products, the fourth generation of ECs comprises a stack powered by batteries with set voltage and various forms, such as USB or teardrop [27].

## 2. Constituents of E-Cigarettes

By heating a liquid that typically has many ingredients, e-cigarettes make an aerosol. Individuals breathe this aerosol into their lungs, when a user exhales into the air, this aerosol might also be inhaled by onlookers. Vapers produce an aerosol made up of a mixture of liquid droplets. It is reported that the aerosol size in the indoor environment is smaller than 50 nm [28]. Due to the large surface area of the alveolar region’s airways, a significant proportion of inhaled e-cigarette aerosol is anticipated to infiltrate and deposit in the deep lung. The deposited dose is the antecedent of the internal dose before considering respiratory clearance and absorption rate. As a result, the amount of e-cigarette aerosol that is deposited may be the best indicator of the health risks associated with passive vaping. However, because of the shortcomings of traditional experimental techniques, previous research has depended on numerical models or a partial human airway replica [17,28].

### 2.1. E-Cigarette Liquids

E-Liquids, also known as vape juice or e-juice, are the liquid solutions that are used in electronic cigarettes (ECs) to produce vapor [29]. It typically consists of glycerol and propylene glycol, flavors, nicotine, formaldehyde, and other chemicals that are heated, aerosolized, and inhaled [30]. A new body of evidence indicates that e-liquids often contain a variety of potentially toxic chemicals [31]. Propylene glycol (PG) and Vegetable glycerin (VG) are both used as carriers for the flavorings and nicotine in e-liquids. Propylene glycol is a thinner liquid that produces a stronger throat hit and a more intense flavor, while VG is a thicker liquid that produces more vapor and a sweeter taste. The ratio of PG to VG in an e-liquid can affect the overall flavor, throat hit, and vapor production [32,33]. Flavorings are added to e-liquids to provide a wide range of tastes and aromas [34]. Some common flavors include fruit, candy, dessert, and menthol. Some e-liquids also contain nicotine, which is an addictive substance found in tobacco as shown in Table 1. The concentration of nicotine in an e-liquid can vary, and users can choose from a range of strengths to suit their preferences [35,36]. It is important for users to be aware of the ingredients in their e-liquids and to choose high-quality products from reputable manufacturers. Poor-quality e-liquids can contain harmful contaminants or incorrect levels of nicotine, which can be dangerous. It is also important to store e-liquids safely, away from children and pets, and to follow the manufacturer’s instructions for use [37]. According to the American Cancer Society, Formaldehyde: This is a carcinogen that can form when e-liquid overheats or when not, enough liquid reaches the heating element (known as “dry puff”) [38,39].

#### Flavors of E-Liquids

E-flavors, also known as flavorings, are the compounds used to give electronic cigarette (EC) e-liquids their taste and aroma. E-Flavors come in a wide range of flavors and can be mixed and matched to create unique combinations as shown in Table 2. Some popular flavors include fruit, candy, dessert, and menthol. Some e-liquid manufacturers also offer tobacco flavors for users who want a more authentic smoking experience [40].

### 2.2. E-Cigarette Aerosol

Electronic cigarette (EC) aerosol, also known as vapor, is the cloud of particles that is produced when e-liquid is heated and turned into a gas. EC aerosol typically consists of a mixture of water, propylene glycol, vegetable glycerin, flavorings, and sometimes nicotine [41]. The composition of EC aerosol can vary depending on the specific e-liquid used and the device used to heat it. The amount and size of the particles in the aerosol can also be affected by factors such as the voltage of the device, the temperature of the heating element, and the user’s inhaling technique [42]. While EC aerosol is generally considered less harmful than the smoke produced by traditional cigarettes, it is not completely harmless [43]. An e-cigarette does not burn tobacco but produces an aerosol. Cigarette smoke, on the other hand, has thousands of known toxic and possibly dangerous elements, whereas the composition of EC aerosol is significantly less complex. however, at significantly lower levels than in cigarette smoke [44,45]. According to gravity measurements, glycerol, propylene glycol, water, and nicotine made up 89–99% of the e-cigarette aerosol composition, with additional, minor components making up about 3% [46].

## 3. Chemicals Used in E-Liquids

Contrary to common assumption, electronic nicotine delivery systems do not often burn tobacco, which prevents the release of dangerous compounds. Instead, the high temperatures (>200 °C) that are achieved by e-cigarette solutions are tobacco-specific. acetaldehyde, a possible carcinogen, metals, nitrosamines, and carbonyl compounds including acrolein and formaldehyde, which are human carcinogens, according to the International Agency for Research on Cancer [47,48,49,50,51]. Although the quantities produced by e-cigarettes are lower than those in tobacco smoke, they are nevertheless adequate to contribute to carcinogenesis because they contain the recognized carcinogens formaldehyde and acrolein. It is yet unknown whether lower carcinogen levels have been seen in EC users as compared to smokers [52].

### 3.1. Carbonyl Compound

#### 3.1.1. Formaldehyde

**Figure 1:**
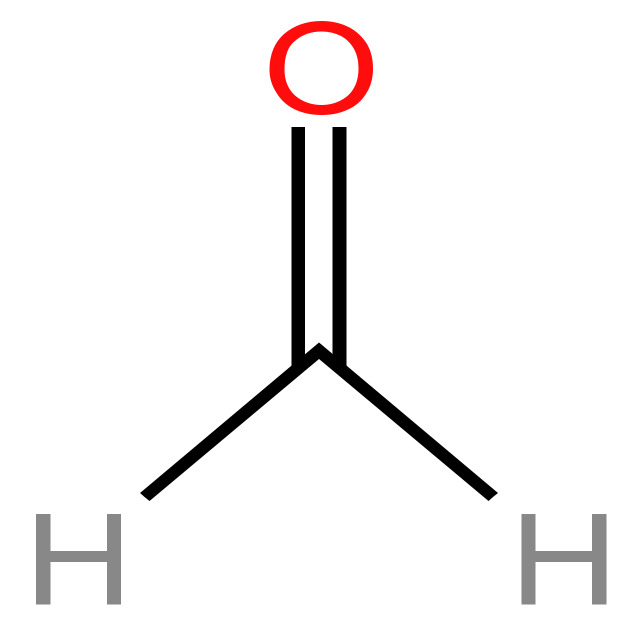
Chemical structure of formaldehyde [53].

Formaldehyde-containing hemiacetals were observed to be detectable by Nuclear magnetic resonance spectroscopy during the evaporation process. Its chemical structure is shown in Figure 1. Propylene glycol and glycerol are known formaldehyde releasers. Formaldehyde-releasing compounds averaged 38,090 g per sample (10 puffs) at high voltage (5, 0 V), according to an analysis of a commercial e-liquid used in a “tank system” e-cigarette with a variable voltage battery. It might accumulate in the respiratory system more quickly than gaseous formaldehyde, which might increase the likelihood of developing cancer. This risk is five times greater than the risk of chronic smoking [53].

#### 3.1.2. Acetaldehyde

**Figure 2:**
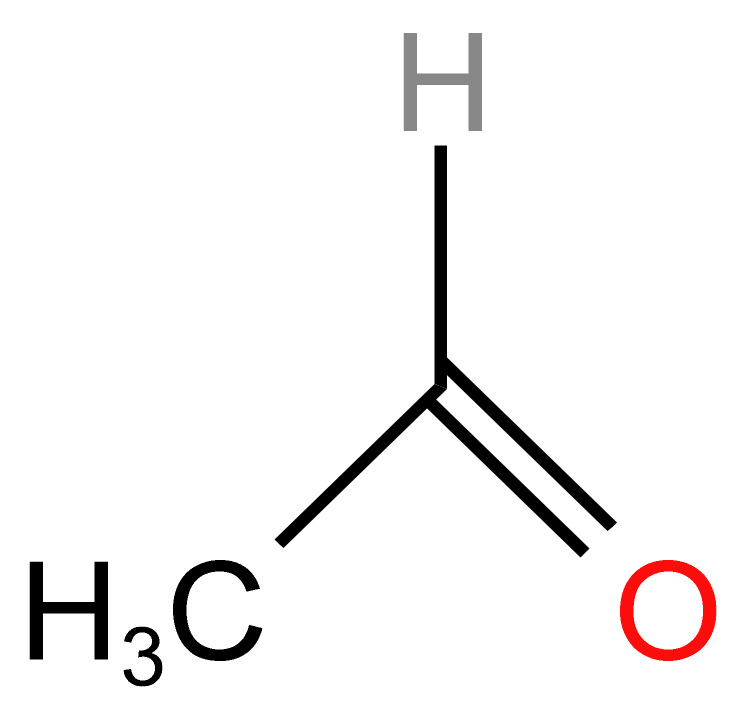
Chemical Structure of Acetaldehyde [54].

Acetaldehyde has been classified by the Institute of Medicine as the most important cardiovascular (CV) toxin in tobacco smoke. Its chemical structure is shown in Figure 2 [54]. Acetaldehyde is found in tobacco smoke (700–800 μg/cigarette in regular smoking) Contained in cigars and water pipes (hookah and hookah) and is also found in e-cigarette (e-cigarette) aerosols [53]. The risk of cancer from acetaldehyde exposure may be particularly significant for individuals who use ECs over the long term, as repeated exposure to the chemical can lead to the accumulation of DNA damage and other cellular changes that increase the risk of cancer. Studies have shown that the levels of acetaldehyde in EC aerosols can vary widely depending on the type of EC device, the power setting, and other factors [55]. However, even at low levels, acetaldehyde has been shown to have carcinogenic properties and has been linked to an increased risk of cancer, particularly in the upper respiratory tract and the head and neck area [56]. Acetaldehyde is a carcinogen and may promote cancer development through multiple mechanisms, including interfering with DNA replication, inducing DNA damage, and forming DNA adducts [57].

#### 3.1.3. Acrolein

**Figure 3:**
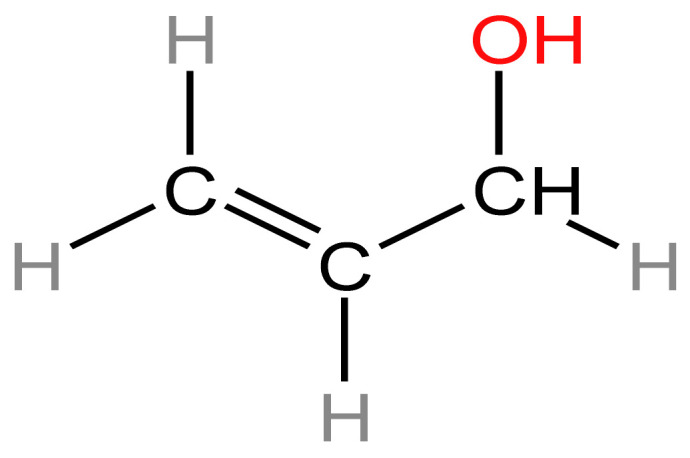
Chemical structure of acrolein [54].

Acrolein has been classified by the Institute of Medicine as the most important cardiovascular (CV) toxin in tobacco smoke. Its chemical structure is shown in Figure 3 [54]. Acrolein is a toxic chemical that is present in both tobacco smoke and e-cigarette aerosol. It is formed when glycerin, a common ingredient in e-liquids, is heated during the vaping process. Acrolein is a known respiratory irritant and can damage DNA, which can lead to cancer. Several studies have investigated the potential cancer risk associated with acrolein exposure from e-cigarette use. Some studies have suggested that e-cigarette aerosol may contain levels of acrolein that are higher than those found in tobacco smoke. One study found that acrolein levels in e-cigarette aerosol were up to 14 times higher than those found in tobacco smoke [53,55,56,57]. Acrolein was also found to form DNA adducts in TP53 mutational hotspots similar to those found in smoking-related lung cancers, suggesting that acrolein may be a relevant etiologic agent involved in her E-cigarette smoking. increased. Numerous studies have demonstrated that chronic exposure to acrolein promotes cardiovascular disease (CVD), whereas even low-level acute exposure to the substance causes dyslipidemia, vascular damage, endothelial dysfunction, and platelet activation. Acrolein is involved in the development of cancer, according to investigations on animals [58,59,60,61,62,63,64,65].

### 3.2. Alcohol

#### 3.2.1. Menthol

**Figure 4:**
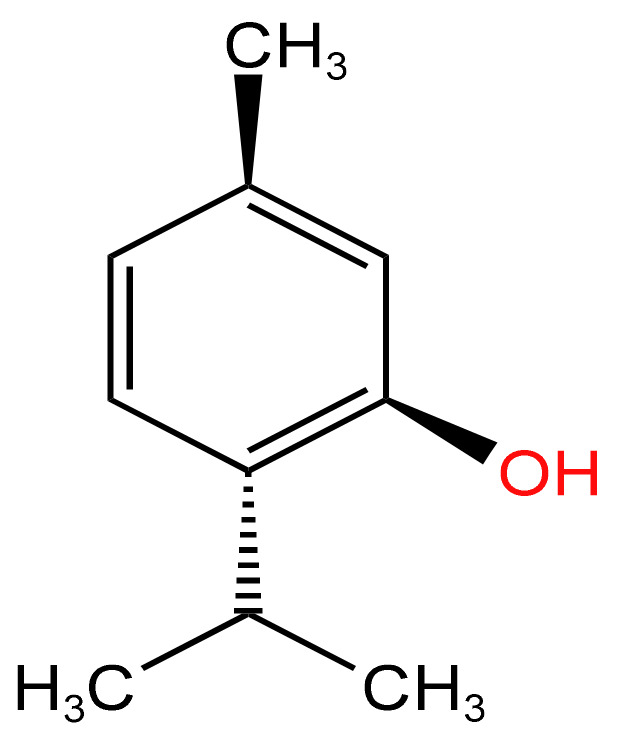
Chemical structure of menthol [65].

Modulation of nicotine metabolism and direct carcinogenic/pro-inflammatory effects are the two main mechanisms by which menthol exerts its potentially cancer-causing effects. Figure 4 depicts its atomic structure. Menthol is also linked to nicotine, both indirectly (via direct effects on endogenous responses to nicotine, such as through modulation of nicotinic receptor expression) and directly (through greater tolerance/reduced throat irritation of TS [ECS]). It is linked to an overall rise in the prevalence of addiction [65]. When exposed to various aromatic ELs, including those with menthol as an ingredient, lung cancer cells’ ability to invade and metastasize was demonstrated to increase, according to another study by Zahedi [66].

#### 3.2.2. Ethyl maltol

**Figure 5:**
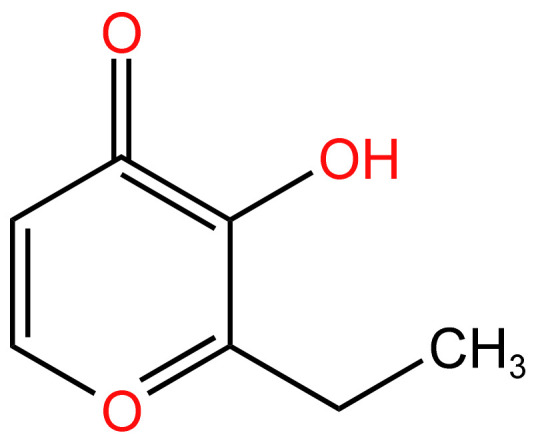
Chemical structure of ethyl maltol [67].

Foods frequently include ethyl maltol (EM), a flavoring ingredient that is regarded as generally harmless. It has been found in the aerosols of numerous commercial e-cigarette vaping fluids. This work investigates whether EM increases heavy metal-mediated toxicity because EM accelerates heavy metal transport across plasma membranes and heavy metals have been found in aerosols produced by e-cigarettes. Further radical generation has been found to come from its interaction with iron and copper, which are typically present in the heating element and/or as impurities. The chemical structure of ethyl maltol is shown in Figure 5. Additionally, it has been shown to promote additional pro-inflammatory effects and enhance systemic exposure to inhaled chemicals, as well as to trigger an inflammatory response, modify local immune function, and damage epithelial barrier function and integrity. This strongly shows that ethyl maltol is carcinogenic given the proven oncogenicity of free radicals both individually and collectively. [67,68].

#### 3.2.3. Ethanol

**Figure 6:**
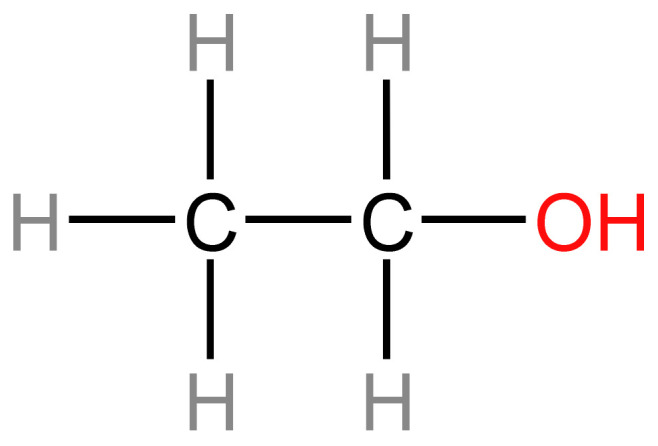
Chemical structure of ethanol [69].

Ethanol is classified as a carcinogen by the International Agency for Research on Cancer. Its structure is shown in Figure 6 [69]. Although there are rules for disclosing substances, including ethanol, in e-liquids in other nations, ethanol has been noted as an undeclared ingredient in nicotine-containing e-liquids sold in the United States. [70]. EtOH alters epigenetics by altering DNA and histone methylation and acetylation. This may affect the regulation of gene expression even after transplacental exposure. On the other hand, the evidence for the carcinogenicity of EtOH in laboratory animals is insufficient. However, no concrete evidence has been found that ethanol in e-liquids can cause cancer [71,72].

### 3.3. Tocopherol 

**Figure 7:**
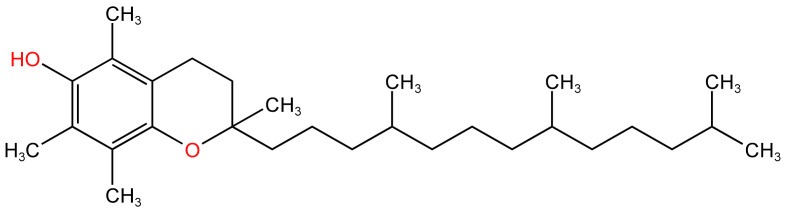
Chemical structure of tocopherol [75].

Vitamin E, especially acetate, is an oily substance at room temperature and is used as both a diluent and thickener in a few tablespoons. In particular, those involving cannabis derivatives [73]. Vitamin E acetate is an oil-based ingredient that was sometimes added to EC e-liquids as a thickening agent. When inhaled, the oil can coat the lungs and cause respiratory problems. Vitamin E acetate has been implicated in a series of recently reported cases of e-cigarette-related lung injury. Its chemical structure is shown in Figure 7 [74,75]. Wu reported that the thermal decomposition of vitamin E produces highly toxic and irritating ketene gas (by removal of the aryl acetate group) and many other toxic and reactive chemicals with detectable carcinogenic activity, including benzene and various alkene [76].

### 3.4. Metals 

**Figure 8:**
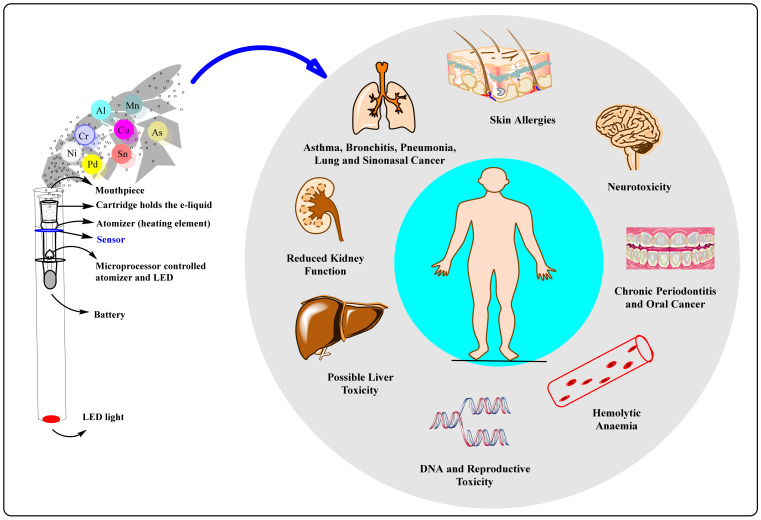
Important parts of e-cigarette and health impacts of releasing trace metals.

According to certain research, dangerous heavy metals like cadmium and lead can be discovered in both cigarette smoke and the aerosol produced by e-cigarettes. Toxic heavy metals like lead, chromium, nickel, manganese, and nickel are present in higher concentrations in e-cigarette aerosols and e-liquids than in cigarettes, according to a new study by the California Department of Public Health (Figure 8) [48,76,77]. Heavy metal exposure, which is included in e-cigarettes, can have detrimental consequences on health. Respiratory conditions like lung cancer have been linked to chromium and nickel in some e-cigarette brands. Compounds of chromium and nickel are employed in welding, electroplating, and other industrial operations. Neurological and developmental issues can result from lead and manganese exposure. Batteries, insecticides, and steel manufacture all employ manganese compounds. Batteries, ammunition, metal items, paints, and ceramics are all made with lead compounds. Cadmium exposure is linked to lung cancer and can harm the kidneys. Nickel-cadmium batteries contain cadmium compounds, which are also utilized in the manufacture of coatings, plastics, ceramics, glass coloring pigments, and polymers. The components in e-cigarettes that raise the risk of cancer are not just heavy metals. In addition to being carcinogens, other compounds including formaldehyde and acetaldehyde also present extra risks to e-cigarette users. In summary, e-cigarettes have a lot of components that could make users more likely to get cancer [76].

## 4. Cancer Caused by E-Cigarettes

The long-term health effects of using electronic cigarettes (ECs) are not yet fully understood, as the technology is relatively new, and research is ongoing. However, there is evidence to suggest that EC use may increase the risk of certain types of cancer. The presence of carcinogens in the body fluids of e-cigarette users inherently means that cells are at risk of oncogenic transformation [78,79]. Nicotine itself may not be a carcinogen, but it is still a highly addictive substance that can have a range of negative health effects, including increased heart rate and blood pressure, constricted blood vessels, and reduced lung function. Nicotine from aerosols or e-cigarette liquids remains on surfaces for weeks or months and reacts with the environment to form nitrates and tobacco-specific nitrosamine compounds, leading to inhalation, ingestion, or dermal contact with carcinogens [80,81].

### 4.1. Head and Neck 

Studies using different brands of electronic vapor with or without nicotine, as well as heavy metals like cadmium, lead, nickel, and nitrosamines showed decreased cell viability and apoptosis compared to unexposed controls and significant evidence of necrosis in head and neck squamous cell carcinoma and normal epithelial cell lines. Additionally, exposed cell lines expressed increased H2A histone family member X (-H2AX), a recognizable indicator of double-stranded DNA breakage [82].

In a case report, Korrapati A. et al., 2016 [83] reported significant DNA double-strand breaks being induced in cells exposed to e-cigarette (0.5–2% volume e-cig vapor over from 24 h to 4 weeks) aerosols as well as an increase in the migration of HN cancer cells after e-cigarette treatment with upregulation of EMT-promoting genes.

The case report by Nguyen et al., 2017 [84] describes a 59-year-old man who developed a basaloid squamous cell carcinoma after using 30 e-cigarettes every day for the previous 13 years. The authors suggest that e-cigarette use may have contributed to the development of basaloid squamous cell carcinoma, highlighting the potential risks associated with long-term e-cigarette use.

The case report by Nguyen et al., 2017 [84] describes a 66-year-old man who developed a basaloid squamous cell carcinoma after using e-cigarettes every day for the previous 13 years, 20 times. The authors suggest that e-cigarette use may have contributed to the development of basaloid squamous cell carcinoma, highlighting the potential risks associated with long-term e-cigarette use.

The case report by Klawinski et al., 2021 [85] describes a 19-year-old man who developed a nonhealing left lateral tongue ulcer later found as a stage IV tumor after using e-cigarettes (0.5 packs) each day for four years. The patient used vaping daily nicotine-delivery systems (Juul) and had no history of tobacco smoking. The authors suggest that e-cigarette use may have contributed to the development of cancer, highlighting the potential risks associated with long-term e-cigarette use.

### 4.2. Lungs

Other cancer-causing substances found in e-cigarette aerosols or vapor include formaldehyde, toluene, acetaldehyde, and acrolein as well as heavy metals including cadmium, lead, nickel, nitrosamines, and other substances.

The case report by Fracol et al., 2017 [86] describes a 51-year-old female who developed breast cancer after using e-cigarettes. Since she believed e-cigarettes were safer than regular cigarettes, she switched to them around three months before her operation and continued to use them at a rate equivalent to her previous 1.5 packs per day. The authors suggest that e-cigarette use may have contributed to the development of breast cancer, highlighting the potential risks associated with long-term e-cigarette use.

A case report by Madsen et al., 2016 [87] demonstrates that e-cigarette usage (38 mg/mL, 10 mL per week), known as vaping, caused severe liver and lung inflammation in a 45-year-old patient, simulating metastatic disease. Results showed that e-cigarette use promoted Epithelial-mesenchymal transitions (EMT) translocation and interfered with DNA repair mechanisms, which supported the link between e-cigarette use and the progression of cancer.

In the case study by Aherrera et al., 2017 [88] it was discovered that e-cigarettes contain nicotine and its metabolites as well as a small amount of nickel in the users’ saliva, urine, and exhaled breath. Surprisingly, although the customers do not smoke tobacco, they nevertheless run the danger of developing lung cancer from the nicotine and nickel in e-cigarettes.

### 4.3. Bladder

E-cigarette liquids have been found to include aromatic amines, aldehydes, and polyaromatic hydrocarbons, all of which have been found to cause bladder cancer in humans. According to a recent assessment by the National Academy of Sciences, there is currently no evidence connecting e-cigarette usage to the onset or prognosis of cancer, however, e-cigarettes do cause the inhalation of carcinogenic substances. The data indicate that is still growing.

A case report by Fuller et al., 2018 [89] describes a man aged 39.4 years smoking for 19.9 years. With a wide range of formulas, e-cigarettes have historically been uncontrolled. Benz(a)anthracene and benzo(a)pyrene, aromatic amines, and aldehydes, among other bladder carcinogens, have been found in e-cigarette liquids, vapor, or urine in previous research. The levels of the bladder cancer-causing chemicals 2-naphthylamine and o-toluidine in the urine of e-cigarette users are higher when compared to non-smoking, non-e-cigarette-using controls. Before supplying samples for this investigation, the majority of these subjects had not smoked a typical cigarette in over a year. Although there is evidence to suggest that e-cigarettes are safer than conventional cigarettes, the current study raises the possibility that using an e-cigarette with varying liquid and vapor control formulas may not be completely risk-free from the perspective of bladder cancer.

The case report by Viswam et al., 2018 [90] describes a 16-year-old girl who developed hypersensitivity pneumonitis after using e-cigarettes for several months. The patient presented with symptoms such as cough, shortness of breath, and fever, and was diagnosed with hypersensitivity pneumonitis based on clinical and radiographic findings. The authors suggest that e-cigarette use may have contributed to the development of hypersensitivity pneumonitis in this patient, highlighting the potential risks associated with e-cigarettes on lung health. To fully comprehend the effects of e-cigarettes, use on lung health, more research is required.

One of the first studies by Lee et al., 2018 [91] showed that the nitrosamines and downstream metabolites of nicotine present in ECs put E-cigarette users at greater risk than non-users for developing lung or bladder malignancies or heart disease.

The case report by Bjurlin et al., 2021 [92] shows in contrast to non-e-cigarette users, those who use e-cigarettes have higher levels of carcinogens that can be metabolized into several compounds that can cause bladder cancer, which can be identified by urine sampling.

### 4.4. Breast

Breast cancer (BC) is the cancer that affects women most frequently in the United States, accounting for almost one-third of all cancer diagnoses in this population and more than 18–20% of all cancer-related deaths in women. There is evidence that e-cigarettes promote lung metastasis of human breast cancer cells. This is an important contribution to understanding the potential risks that e-cigarettes pose to human health and requires further research [93].

Previous research has shown that e-cigarette use increases lung carcinogenesis by causing the production of DNA adducts in the lungs. Additionally, a case report by Ryu et al., 2018 [94] demonstrates that inhaling e-cigarettes may cause the release of oncogenic cytokines or microRNA from both pulmonary cells and breast cancer cells, promoting lung colonization of breast cancer cells like the colonization of conventional CS cells, which promotes the metastasis of breast cancer-causing breast cells.

A study by Kien Pham et al., 2020 [95] in the context of e-cigarette-enhanced BC development and metastasis, evaluates the crucial involvement of myeloid cells and related signaling pathways. The microenvironment of every organ in the body is typically tumor-suppressive under physiological circumstances. However, a tumor-promoting microenvironment can develop as a result of persistent inflammation brought on by a variety of causes.

In a study by Hyunh et al., 2020 [96] e-cigarette inhalation, similar to conventional cigarettes, may induce the release of oncogenic cytokines or microRNAs from both lung and breast cancer cells, thereby promoting lung colonization by breast cancer cells. A third possibility is that exposure to e-cigarettes may improve the survival of breast cancer cells during the invasion and nesting process. Evidence from the literature suggests that cancer cells are prone to apoptosis during metastasis.

## 5. Conclusions

While the long-term health effects of e-cigarette use are still being studied, there is evidence to suggest that e-cigarette use may increase the risk of cancer as well as other diseases like cardiovascular and respiratory disease, due to the potential for harmful chemicals and flavorings in the aerosol. Therefore, it is important to exercise caution when using e-cigarettes and to consider alternative methods for smoking cessation. The safest course of action is to avoid using e-cigarettes altogether. In addition to the potential cancer risk associated with e-cigarette use, there are other health concerns to consider. E-cigarettes contain nicotine, which is addictive and can harm brain development in adolescents. Nicotine can also raise blood pressure, increase heart rate, and constrict blood vessels, which can increase the risk of heart disease. Overall, while e-cigarettes may have very few benefits as a smoking cessation tool, the risks associated with their use, including the potential for cancer, should be carefully considered before use. However, any substance breathed over an extended period of time may be harmful to the lungs, according to the European Respiratory Society [97].

## Figures and Tables

**Table 1:** A List of different marketed e-cigarettes *.

S. No.	Brand Name	Company Name	Composition	Introduced Year
1.	SUORIN	FOXCONN TECHNOLOGY GROUP	Propylene glycol, vegetable glycerin, nicotine, and flavorings	2017
2.	JUUL	JUUL Labs	Propylene glycol, glycerol, flavorings, nicotine	2015
3.	GREEK VAPE	GREEKVAPE	Propylene glycol, glycerol, flavorings, nicotine	2015
4.	LOST VAPE	LOST VAPE	Propylene glycol, glycerol, flavorings, nicotine	2014
5.	MARKTEN	ALTRIA GROUP	Propylene glycol, glycerol, flavorings, nicotine	2013
6.	ASPIRE	ASPIRE VAPE CO. LTD.	Propylene glycol, vegetable glycerin, nicotine, and flavorings	2013
7.	VUSE	REYNOLDS AMERICAN	Propylene glycol, glycerol, flavorings, nicotine	2013
8.	VYPE	BRITISH AMERICAN TOBACCO	Propylene glycol, vegetable glycerin, nicotine, and flavorings	2013
9.	EONSMOKE	EONSMOKE LLC	Propylene glycol, vegetable glycerin, nicotine, and flavorings	2011
10.	INNOKIN	SHENZHEN INNOKIN TECHNOLOGY CO. LTD.	Propylene glycol, vegetable glycerin, nicotine, and flavorings	2011
11.	SMOK	SHENZEN IVPS TECHNOLOGY CORPORATION	Propylene glycol, glycerol, flavorings, nicotine	2010
12.	LOGIC	JAPANTOBACCO INTERNATIONAL	Propylene glycol, glycerol, flavorings, nicotine	2010
13.	HALO	NICOPURE LABS	Propylene glycol, vegetable glycerin, nicotine, and flavorings	2009
14.	VAPORESSO	SMOORE	Propylene glycol, vegetable glycerin, nicotine, and flavorings	2009
15.	Blu	FONTEM VENTURES	Propylene glycol, vegetable glycerin, nicotine, and flavorings	2009
16.	JOYETECH	JOYETECH CO. LTD.	Propylene glycol, vegetable glycerin, nicotine, and flavorings	2007
17.	NJOY	NJOY LLC	Propylene glycol, glycerol, flavorings, nicotine	2006

* [29,35,40].

**Table 2:** A List of different e-liquid flavours ^#^.

S. No.	E-Liquid Flavor	Flavor Profile	Company	Nicotine Strength	Size (mL)
1.	Blue Raspberry	Sweet and fruity	Juul Labs	5%	1.8
2.	Tobacco	Traditional tobacco flavor	Vuse	3%	3
3.	Mango	Sweet and tropical	Puff Bar	5%	1.3
4.	Menthol	Cool and refreshing	Blu	2.4%	2
5.	Vanilla Custard	Creamy and rich	Halo	6 mg	30
6.	Watermelon	Juicy and refreshing	Njoy	4.5%	2.4
7.	Cinnamon Roll	Warm and spicy	Vapetasia	3 mg	60
8.	Strawberry Milkshake	Creamy and fruity	Dinner Lady	12 mg	10
9.	Lemon tart	Lemon curd and pastry	Dinner Lady	3 mg	10
10.	Killer kustard	Vanilla custard	Vapetasia	3 mg	60
11.	Red astaire	Blackcurrant, aniseed, and menthol	T-Juice	3 mg	10
12.	Heisenberg	Blue raspberry, grape, and menthol	Vampire Vape	3 mg	10
13.	Pink lemonade	Pink lemonade	Element E-Liquid	3 mg	20
14.	Hawaiian pog	Passionfruit, orange, and guava	Naked 100	3 mg	60
15.	Black ice	Blackcurrant, aniseed, and menthol	Zeus Juice	3 mg	50
16.	Melon Twist	Watermelon and honeydew	Twist E-Liquids	3 mg	60
17.	Tribeca	Tobacco and caramel	Halo	3 mg	10
18.	Milkman	Vanilla bean ice cream and fruit tart	The Milkman	3 mg	30
19.	Blue Slushie	Blue raspberry slushie	Keep It 100	3 mg	100

^#^ [39,40].

## Data Availability

Data can be made available on request.
